# Efficient, Hierarchical, and Object-Oriented Electronic Structure Interfaces for Direct Nonadiabatic Dynamics Simulations

**DOI:** 10.1021/acs.jctc.5c00878

**Published:** 2025-09-10

**Authors:** Sascha Mausenberger, Severin Polonius, Sebastian Mai, Leticia González

**Affiliations:** † Institute of Theoretical Chemistry, Faculty of Chemistry, 27258 University of Vienna, Währinger Straße 17, 1090 Vienna, Austria; ‡ Vienna Doctoral School in Chemistry (DoSChem), University of Vienna, Währinger Straße 42, 1090 Vienna, Austria; § Research Platform on Accelerating Photoreaction Discovery (ViRAPID), University of Vienna, Währinger Straße 17, 1090 Vienna, Austria

## Abstract

We present a novel, flexible framework for electronic structure interfaces designed for nonadiabatic dynamics simulations, implemented in Python 3 using concepts of object-oriented programming. This framework streamlines the development of new interfaces by providing a reusable and extendable code base. It supports the computation of energies, gradients, various couplings—like spin–orbit couplings, nonadiabatic couplings, and transition dipole moments—and other properties for an arbitrary number of states with any multiplicities and charges. A key innovation within this framework is the introduction of hybrid interfaces, which can use other interfaces in a general hierarchical manner. Hybrid interfaces are capable of using one or more child interfaces to implement multiscale approaches, such as quantum mechanics/molecular mechanics where different child interfaces are assigned to different regions of a system. The concept of hybrid interfaces can be extended through nesting, where hybrid parent interfaces use hybrid child interfaces to easily setup complex workflows without the need for additional coding. We demonstrate the versatility of hybrid interfaces with two examples: one at the method level and one at the workflow level. The first example showcases the numerical differentiation of wave function overlaps, implemented as a hybrid interface and used to optimize a minimum-energy conical intersection with numerical nonadiabatic couplings. The second example presents an adaptive learning workflow, where nested hybrid interfaces are used to iteratively refine a machine learning model. This work lays the groundwork for more modular, flexible, and scalable software design in excited-state dynamics.

## Graphical abstract

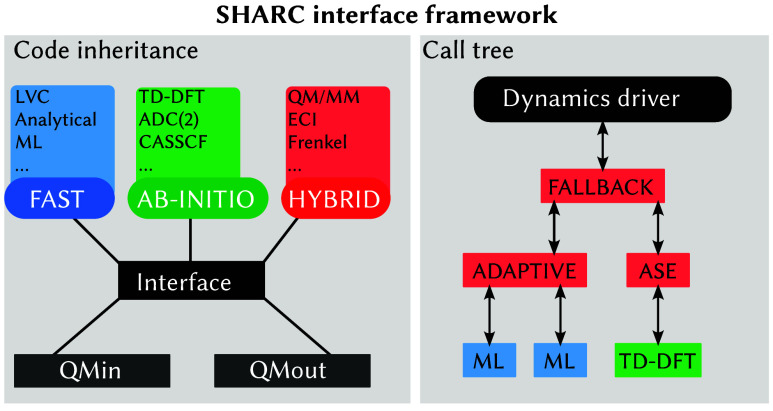

## 1 Introduction

Nonadiabatic molecular dynamics simulations are essential for elucidating the photophysical and photochemical behavior of molecular systems.^1,2^ This need has driven the development of many nuclear dynamics techniques,^1–3^ ranging from fully quantum dynamics approaches—such as multiconfigurational time-dependent Hartree (MCTDH)^4^—to hybrid quantum-classical methods, such as ab initio multiple spawning (AIMS)^5^ and its variants,^6^ as well as the widely employed trajectory surface hopping (TSH) method.^7,8^ Many of these methods follow the “direct dynamics” or “on-the-fly” approach,^5,6,8,9^ where the electronic energies, forces, and couplings are computed locally at each time step along the nuclear/electronic propagation. For most dedicated nonadiabatic dynamics packages, the computation of electronic quantities at every time step is performed externally by another software, e.g., an electronic structure code. Examples of dedicated nonadiabatic dynamics packages are Quantics,^9^ PySpawn,^10^ Newton-X,^11^ NEXMD,^12^ JADE,^13^ Libra,^14^ PYXAID,^15^ as well as the SHARC (surface hopping including arbitrary couplings) package developed in our group.^16,17^

An efficient communication between the electronic structure provider and the nonadiabatic dynamics package is thus a critical component of any direct dynamics simulation. To that purpose, most nonadiabatic dynamics packages include a set of *interfaces* that receive the current nuclear coordinates from the dynamics driver, communicate with the external electronic structure code, and return the obtained results in a well-defined format to the dynamics driver.^11,18–22^ In this approach, it is principally possible to exchange the interface in order to use different electronic structure providers with the same dynamics code. Historically, our^17^ and other nonadiabatic dynamics packages^11,13^ used interfaces that communicate with the dynamics driver via file I/O and that reinitialize the electronic structure calculation in each time step. For fast electronic structure methods (e.g., semiempirical methods,^23^ time-dependent density tight binding,^24^ vibronic coupling models,^25^ or machine learning models^26^), the repeated file I/O and reinitializing constitutes a massive computation overhead in the dynamics. For this reason, recent developments in nonadiabatic dynamics packages have focused on improving the efficient communication between the driver and the interface.^27–30^ A pioneering example of these implementations is PySHARC,^27^ which links the SHARC dynamics code via C code to the Python-based interfaces. This technique allows for extremely efficient TSH simulations when combined with fast methods such as linear vibronic coupling (LVC) or machine learning potentials, exhibiting computational costs of few CPUh per picosecond, even for large transition metal complexes with tens of electronic states,^31^ and nanosecond simulation times.^32^

While computational efficiency is one very important factor when developing interfaces, three other critical aspects are the maintainability, extensibility, and reusability of the code, as well as the flexibility that users have when using the interfaces. The first aspect is related to the fact that for particular problems in nonadiabatic dynamics, specific electronic structure methods might be needed. Hence, it should be desirable to develop new interfaces or to extend existing ones in an straightforward manner. This is also related to recent thrusts in the community toward more reusable and standardized code^33,34^ that improves interoperability. The flexibility aspect becomes particular important when dealing with large systems or slow dynamics. For example, simulating large multichromophoric chemical systems^35^ is particularly challenging due to their size and complexity, and fragment-based (”divide and conquer”) methods offer a promising strategy to reduce computational costs and improve scalability.^36–39^ Such a setting requires code that allow users to divide the system into fragments as needed, apply the most suitable electronic structure methods to each fragment, and then seamlessly combine them into the full system—capabilities that go beyond what typical interfaces can provide. Another setting where straightforward nonadiabatic dynamics simulations are not sufficient are cases involving slow dynamics due to long-lived excited states. In these situations, accelerated dynamics techniques^40^ or sampling the crossing seam space^41,42^ can provide more insight than brute-force time propagation. Also the increasing integration of machine learning methods into nonadiabatic dynamics^43–46^ requires efficient means of obtaining well-sampled training data. These scenarios underscore the critical need of flexible and adaptable workflows within nonadiabatic dynamics simulations.

In response to these challenges, here we introduce a novel interface framework for nonadiabatic dynamics simulations. The three primary features of this framework are (i) efficient communication with a dynamics driver, (ii) ease of code maintenance, reuse, and extendability, and (iii) hierarchical communication among interfaces. In addition to these primary objectives, the framework is designed—unlike comparable implementations^47–50^ —to support an arbitrary number of states with arbitrary multiplicities and charges, as well as simultaneous computation of multiple properties, such as more than one gradient, nonadiabatic couplings, and transition moments. The interface framework is implemented in Python and advances previous SHARC and PySHARC interfaces,^17,27^ which already fulfill goal (i). Although the interface framework is an integral part of SHARC (code and extensive documentation are available in the SHARC 4 release^16^ under the GNU General Public License), it is designed as a modular part of the code, making it easily adaptable for integration into other nonadiabatic dynamics packages. The implementation makes heavy use of object orientation with inheritance, allowing to reuse a large fraction of code in multiple interfaces, simplifying maintenance, and cutting down the development time for new interfaces (goal (ii)). While object-oriented programming was employed in several ground state dynamics implementations,^51,52^ so far it has not obtained much attention in nonadiabatic dynamics simulations.^48^ However, the most innovative part of the new interface framework is the introduction of *hybrid* interfaces, which can call other interfaces in a *nested interface call tree*. This design allows for organically delegate tasks, enabling multiscale simulations and highly customizable workflows tailored to the user’s need without the need for user-implemented code (goal (iii)). None of the nonadiabatic dynamics packages mentioned above^10–15,53^ provide such a nestable, user-level interface concept; to the best of our knowledge, the only package with a comparable concept is Cuby,^54^ which, however, lacks excited-state capabilities.

The flexibility of our interface framework has been implicitly exploited in recent quantum mechanics/molecular mechanics (QM/MM) simulations using preparameterized potentials,^55^ and excitonic configuration interaction (ECI) computations.^38^ However, it will be explicitly showcased here with the optimization of a minimum-energy conical intersection using numerical nonadiabatic coupling vectors with time-dependent density functional theory (TDDFT), and a fully automatized workflow for active learning simulations.

## 2 Methodology

This section provides first a brief overview over the necessary communication in nonadiabatic dynamics simulations for contextualization. Next, it describes the new data classes that handle all data processed in the interfaces in a standardized and structured way, followed by the main specifications of the new interface base classes. The two data classes and the four base classes constitute the scaffold for the new interface framework, from which all actual interfaces are derived. We note that here we focus on the general design principles of the new interface framework rather than the code itself or finer implementation details. The documentation of the SHARC 4 package^16^ provides user manuals for all existing interfaces as well as a developers guide and skeleton code to aid the development of new interfaces.

### 2.1 Overview

As stated above, many nonadiabatic dynamics codes are divided into a dynamics driver (the code that handles the propagation of electronic and nuclear degrees of freedom) and the interface code. How they communicate depends primarily on the kind of information that needs to be exchanged. Here, we exemplarily describe the case for the TSH method as implemented in the SHARC package;^56^ however, other methods and packages handle very similar information.

The evolution of the electronic wave function depends on the total electronic Hamiltonian matrix, containing the electronic energies and all relevant coupling terms (e.g., spin–orbit couplings or electric dipole–electric field interactions). The solution of the electronic equation of motion further requires (with exceptions^57^) either nonadiabatic coupling vectors or wave function overlaps,^58^ ideally including full phase information.^59^ Additionally, to solve the nuclear equation of motion, in TSH one requires the gradients of some or all of the electronic states.^56^ Lastly, often it is convenient to compute additional arbitrary data that does not affect the dynamics but is stored for subsequent analyses (e.g., wave function descriptors or ionization probabilities).

Within SHARC, this large set of electronic structure data is provided to the dynamics driver by the interfaces in response to an “interface call” (thus, the driver is referred to as a “caller” below in some instances). In such a call, the driver-provided input consists of the molecule (elements, coordinates, number of states per multiplicity, total charge per multiplicity) and the set of *requests* that tells the interface which electronic quantities are needed. Other input (e.g., basis set, active space, or density functional) is interface-specific and hidden from the driver. This strict separation between the data known by the driver and by the interface is an important design feature of the interfaces presented in this work, as separation facilitates modularity and interoperability.

The new interface framework is written in Python 3 in an object-oriented fashion. We use the concept of inheritance, which allows classes to reuse and extend code from base classes. Through inheritance, a derived class automatically gains the attributes and methods of its base class. This ensures consistent behavior across related classes. Additionally, a derived class can modify or add methods to implement specialized behavior while preserving the overall structure of the base class. Inheritance minimizes the amount of repeated code and facilitates efficient code maintenance. Two data classes—one for all input information and one for the electronic quantities to be returned—are the foundation of the implementation. A general interface base class lays out the strict rules to which all interfaces have to adhere, as described in detail below. Three derived base classes expand the general base class and add code that is useful for certain types of interfaces. Compared to the interfaces that were used in SHARC in the past, the new framework imposes a relatively strict structure to the code. While this somewhat limits the freedom of the developer of an interface, it also simplifies creating a new interface because many required routines are already present and only the interface-specific code needs to be written.

Besides simplifying the development, the strict interface specifications also facilitate that each interface can be directly called from within Python. This makes new interfaces directly usable in a file-I/O-free fashion for improved performance. Furthermore, and perhaps most interesting, the new interface framework makes it possible that a “parent” interface (another example of a “caller”) calls other “child” interfaces, a concept that we shall denote as a *hybrid interface*. This name is chosen because hybrid interfaces facilitate hybrid methods (e.g., hybrid QM/MM) that use different child interfaces for different regions of the system. However, the concept is even more versatile because child interfaces can also be hybrid interfaces and possess their own children. Such nesting of interfaces enables to setup complex workflows with little or no effort of actually modifying the source code, as we will detail below.

### 2.2 Data Classes

Here the new structures used for handling data in the interfaces are discussed. Figure 1 provides an overview over the most important information pertinent to each class.

**Figure 1:**
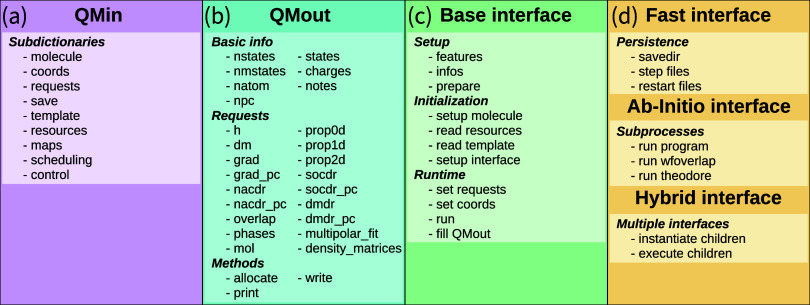
Overview over the organization and data content of the code classes of the SHARC interface framework. (a) The QMin class collects all input information needed into several subdictionaries. (b) The QMout class contains the basic dimensions of the system, the results of any requests (e.g., the Hamiltonian matrix, the array of dipole moment matrices), and methods for the initialization of the object and reading from/writing the data to a file. (c) The SHARC interface base class defines the most important routines of all interfaces for initialization, for all operations carried out at each time step (i.e., at runtime), and for setting up the interface-specific input files. (d) From the base class, three families of interfaces are derived. Fast interfaces have code that allows storage of data that would normally written to files in each time step. Ab initio interfaces possess code for running external programs in subprocesses (possibly in parallel) and for running auxiliary code like wfoverlaps or TheoDORE.^60^ Hybrid interfaces contain code to initialize and run a set of child interfaces. Code for functionality mentioned in (c–d) is automatically inherited by all interfaces derived from the respective base class.

#### 2.2.1 QMin Data Class

The QMin object stores all the information required by a SHARC interface to perform its tasks. The data values can be accessed by various keys in a hierarchical manner (as a dictionary of dictionaries in Python jargon) with built-in type checking and automatic casting functionalities. Most subdictionaries in the QMin object are automatically populated by the base classes, reducing the programmer’s workload and removing the need for detailed knowledge of SHARC internals. Another advantage of the fixed structure of the QMin object is that it formalizes the input in a consistent way for all interfaces, which simplifies the interaction between interfaces in hybrid interface schemes. The QMin dataclass contains subdictionaries with the following information sets: *molecule*, *coords*, *requests*, *save*, *template*, *resources*, *maps*, *scheduling*, and *control*. Here, the first four are very strictly regulated, as they only contain information that comes directly from the driver or another caller and are essential for frictionless communication. The template and resources dictionaries contain all interface-specific settings, e.g., basis set, functional, active space, or relativistic effects, and are freely usable by the interfaces. The maps, scheduling, and control dictionaries are optional and only employed by some interfaces, mostly to prepare and schedule calls to quantum chemistry programs.

#### 2.2.2 QMout Data Class

The QMout object is a data class that collects all the requested electronic structure results after they were obtained by the interface, be it from models or from external programs. This data class additionally possesses code (i.e., object methods) to initialize all requested results for the given number of atoms and states, to read/write the results from/to a file, or to print the data for inspection. Further, it is used to return the requested data to the dynamics driver or a parent interface.

The QMout object (and any optional file created from it) is intended to be as self-descriptive as possible. It retains all relevant dimensions of the system, e.g., the number of states per multiplicity, molecular charge per multiplicity, number of atoms, or number of point charges. Within SHARC, the total number of states includes all sublevels of all multiplets—for example, a simulation with 4 singlets and 3 triplets considers a total of 4 + (3 × 3) = 13 states. Therefore, the interface framework also considers explicitly all sublevels, duplicating spin-independent information wherever necessary. The data class is explicitly designed to always cover all possible matrix elements and vectors for all states and their sublevels.

Most attributes of a QMout object hold some resulting electronic data. The Hamiltonian matrix with matrix elements *H*_*ij*_ contains the electronic energies *H*_*ii*_ = *E*_*i*_ and the spin–orbit couplings if pertinent, so that it is an *n*_total_ × *n*_total_ array. The wave function overlaps matrix (i.e., *S*_*ij*_ = ⟨Ψ_*i*_(*t*_0_)|Ψ_*j*_(*t*)⟩) is of the same dimension, whereas the dipole moment tensor (*D*_*ijp*_ = ⟨Ψ_*i*_|μ_*p*_|Ψ_*j*_⟩ with *p* = {*x*, *y*, *z*}) consists of three such matrices. In the specific case of SHARC, four different derivative tensors are currently handled: the list of gradients (size *n*_total_ × *n*_atom_ × 3), the tensor containing the nonadiabatic coupling vectors (*n*_total_^2^ × *n*_atom_ × 3), the derivatives of the spin–orbit couplings (*n*_total_^2^ × *n*_atom_ × 3), and the derivatives of the dipole moment tensor (3 × *n*_total_^2^ × *n*_atom_ × 3). If the system is embedded with point charges, then four additional tensors contain the respective derivatives with respect to the point charge coordinates.

To enable more sophisticated postprocessing as well as fragment-based electronic structure calculations, the interfaces can also communicate information on the electronic densities through the QMout object. To this end, a separate entry contains the information on the atomic basis set (following PySCF conventions^61^), and a second entry can contain the full tensor of all nonzero electron and spin density matrices in the atomic orbital basis. Alternatively, several SHARC interfaces can – rather than passing the electron densities to the caller– obtain an atom-centered distributed multipole expansion by means of a restrained electrostatic potential fit;^55^ the latter represents the Coulombic potential of each state and transition density by a relatively small number of parameters.

Additionally, the QMout object can hold arbitrary electronic structure data that is not intended to directly affect the nonadiabatic dynamics but only computed along the trajectory and stored for a posteriori analyses. Such data can include different contributions to the total energy (e.g., the energy of the MM subsystem in an QM/MM calculation), wave function descriptors of all states from the TheoDORE^60^ package, or Dyson norms between all pairs of states to describe ionization probabilities, to name few examples. Within SHARC, these data are called zero-, one-, and two-dimensional properties, because, for a single time step, these are scalar numbers, vectors (with one entry per state), or matrices (with one element per state pair). Finally, QMout can store arbitrary meta-data that the interfaces are free to append, e.g., information on convergence, iteration count, wave function diagnostics, or computation times.

### 2.3 Interface Base Class

In this and the following sections, we describe the general structure of the new interface code. At the top of the inheritance hierarchy is a base class, from which three derived base classes have been created. Each of the actual, usable interfaces is then built on top of one of the three derived base classes, depending on the type of desired interface.

All new interfaces are derived from the interface base class. This base class defines all essential functionalities of an interface, like creation of the QMin and QMout objects, basic reading of template and resource files, processing of the requests, and a routine that is called if the interface is called as a standalone Python script. Furthermore, the base class defines several abstract methods of an interface object. Abstract methods are routines that are declared to exist in the base class without being actually implemented; these routines must be implemented for each individual interface by the developer. The abstract methods include the interface-specific postprocessing of the requests and template options, scheduling of multiple quantum-chemistry calculations, generating folders with input files, or parsing output files, as well as routines that assist the user in setting up the interface’s own input files. Thus, the base class serves as a guideline for all other interfaces.

An important part of our interface framework is the new concept of a *feature set* that each interface possesses. By returning its feature set, an interface can communicate which electronic structure properties it can provide and which modes of operation it supports. In this way, e.g., the trajectory setup tool of SHARC can cross-check which dynamics options are possible with a chosen interface, e.g., whether nonadiabatic coupling vectors or wave function overlaps can be used to propagate the electronic wave function, how the velocities will be rescaled, or whether spin–orbit couplings are available. The declaration of feature sets is also critically important to facilitate hybrid interfaces, which require the feature sets of their children to dynamically determine their own feature set and adjust their behavior. For example, in a QM/MM calculation, the MM child needs to return the partial charges of all MM atoms, and the QM child needs to consider these point charges in its calculation. As another example, a numerical differentiation interface can easily check which derivatives a given child can return and which derivatives need to be obtained numerically.

In the spirit of object orientation, we have also revised how the input files for each interface are prepared. In previous SHARC versions, there existed several setup scripts for various tasks, e.g., setting up vertical excitation calculations to obtain initial conditions, setting up trajectories, or setting up calculations to parametrize an LVC model.^62,63^ Each of these setup scripts contains thousands of lines of code to take care of interface-specific setup tasks, such as checking and writing template files, preparing parallel computations, or copying initial orbital guesses. Within our new interface framework, this step is much simplified, as the setup scripts do not contain any such code anymore. Rather, they rely on the special functions of each interface class that can be called during the setup process. Each interface provides three routines, one to return its feature set, one to query the user for any needed interface-specific input, and one to prepare a folder with all relevant interface-specific input files. In this way, every interface is responsible for ensuring the consistency of its input files, simplifying the maintenance of the setup scripts, and ultimately, making it easier to add new interfaces. Furthermore, this approach enables the setup of hybrid interfaces—something that was not possible with the previous code because static setup logic struggles to handle flexible, hierarchical interface calls. In the new interface framework, each hybrid interface is responsible for its own input and also for recursively calling the setup routines of its children, until the input for the entire call tree is prepared.

The new interfaces can be invoked in several different ways—either they are executed as standalone scripts by means of its *main* function, or they are initialized and executed from a dynamics driver or a parent hybrid interface. In any case, each interface first goes through several steps of initialization—see Figure 1c—to collect the input that is static. In this way, laborious initialization tasks are carried out only once, rather than at every time step. The first initialization step defines the molecule section of the QMin object (see Figure 1a), i.e., number of atoms, elements, number of states, multiplicities, charges, and point charges (but no coordinates, as those change during runtime). In the next step, the template file is read, providing interface-specific input ranging from basis set, density functional, or active space, to the definition of analytical or machine learning models. Subsequently, the resource file is read, providing necessary paths, environment options, available CPU cores and memory, and options for auxiliary code (e.g., wave function overlaps code or TheoDORE). The distinction between template and resources is historically grown, but generally follows the rule that all trajectories of a project share the same template file but might use different resource files without affecting the results. The last step of the interface initialization performs various forms of checks of the input; in this phase, hybrid interfaces instantiate their set of child interfaces, leading them recursively through the same initialization steps.

Once an interface is fully initialized, it can be used to carry out any number of electronic structure calculations. In each time step, the dynamics driver will first set the current coordinates and provide a list of requests and some additional information (like the index of the current time step). Subsequently, once the *run* routine of the interface is called, the actual calculation will start. The interface might, e.g., create directories in the scratch area, generate input files, call external programs, and call auxiliary programs to obtain wave function overlaps or wave function descriptors. In the last step during runtime, all obtained results are read, parsed, and collected in the QMout object (see Figure 1b). Hybrid interfaces will instead call the run routines of their children, obtain QMout objects from each, and then then assemble their own QMout object. If the interface was called as a standalone script, then as a last step the resulting QMout object is written to a file; alternatively, the QMout object is simply returned to the caller.

In addition to these basic routines for running an interface, the base class provides code concerned with the management of a save directory. Each interface maintains a save directory, where interface-specific data is stored between time steps. This includes files for molecular orbital guesses, files for configuration interaction coefficients to compute wave function overlaps, or coordinates of previous time steps. In the new interface framework, the save directory management from previous SHARC versions has been overhauled, so that at every point in time each file is clearly assigned to a particular time step. The most recent successful time step is stored in a step file for reference. In this way, the interface can dynamically decide—based on the current step requested by the caller—whether the current calculation corresponds to a new time step or a repeated calculation at a previous time step, and whether the previous invocation of the interface was successful. This represents a considerable improvement over earlier SHARC versions, where files were either labeled “old” or “current”, a scheme prone to inconsistency if a computation fails. With the new framework, the timestamp mechanism allows data from previous steps to be retained for later analysis, or to automatically delete them when no longer needed (a form of garbage collection).

One last functionality that is provided by the interface base class is a new logging system. In previous SHARC versions, interfaces are simply providing progress and error information to standard output. With the introduction of the hybrid interfaces, this approach is no longer viable, as multiple interfaces (parent, children, and possibly further descendants) would write concurrently to the same output. The new logging system makes it possible to redirect the output of each interface within the interface tree to separate targets (standard out, files, sockets, etc.), such that output can be clearly assigned to the interface that produced it. Additionally, the logging system provides different verbosity levels to better control the amount of output for debugging, testing, and production runs.

### 2.4 Derived Base Classes

The base interface class described above is kept very general, only including methods that are necessary or useful for the majority of interfaces. Further generic code that is useful only to certain types of interfaces is placed in three *derived base classes*, that we label as *fast* interfaces, *ab initio* interfaces, and *hybrid* interfaces. These derived base classes inherit all attributes and methods from the base interface class and complement them with additional functionality that is summarized in Figure 1d. Like the base interface class, the derived base classes are abstract classes and cannot be instantiated. The actual ready-to-use interfaces are then derived from any of these three derived base classes. For example, the SHARC–LVC interface^55^ follows the inheritance relationship: interface base class → derived base class for fast interfaces → LVC interface. In this way, the LVC interface inherits all code for setup, initialization, run time, and persistance, as given in Figure 1c–d.

#### 2.4.1 Base Class for Fast Interfaces

The base class for fast interfaces provides code for methods that neither rely on file I/O nor on calls to external software. This includes interfaces that deal with preparameterized potential energy surfaces (PES), such as analytical ones,^40^ those derived from vibronic coupling models,^27,55^ or PES obtained from machine learning.^43,44^ All of these methods provide electronic quantities at very low computational cost, and therefore it is important that trajectories using these interfaces suffer as little computational overhead as possible. Therefore, the fast interface base class provides code to manage the save directory content efficiently, i.e., such that all data that would normally be written to the save directory is instead stored in memory. The code otherwise mimics the normal save directory behavior, e.g., assigning each data to a time step, storing the last successful time step, etc. Additionally, the full set of saved data is written to the save directory during the last time step of a run and is fully read in when a trajectory is restarted.

#### 2.4.2 Base Class for Ab Initio Interfaces

The base class for ab initio interfaces contains functionality to launch external programs and to utilize the auxiliary programs wfoverlap^58^ and TheoDORE.^60^ Additionally, it provides logic to schedule multiple calls of the same quantum chemistry program, either for parallelization purposes, or because some quantum chemistry codes cannot provide all required data in one single run (e.g., some packages can only compute one excited-state gradient at a time). Third, the base class provides generic code to generate and handle various density matrices and to perform electrostatic potential fits of electron densities.

#### 2.4.3 Base Class for Hybrid Interfaces

While the base classes for fast and ab initio interfaces to some extent unify and refactor functionality that was already present in previous SHARC versions, the base class for hybrid interfaces provides entirely new functionalities. This base class provides functionality to streamline the instantiation and execution of a set of child interfaces, including code aiding in tracing errors in any of the child interfaces. Furthermore, there are separate queue implementations for fast children (with minimized overhead) and for ab initio children (with support for parallelization and large density matrix tensors).

### 2.5 Example use Cases for Hybrid Interfaces

In the following, we describe few examples of calculation setups that are ideally suited to be performed within hybrid interfaces, which in turn can be derived from the base class for hybrid interfaces (Section 2.4.3). These examples are illustrated as call trees in Figure 2. Call trees describe the communication between the parent interface on top and the child interface(s) on the bottom. Incoming input information (described by the QMin data class) is distributed from top to bottom, whereas outgoing electronic structure results (encapsulated by the QMout data class) are retrieved from bottom to top.

**Figure 2:**
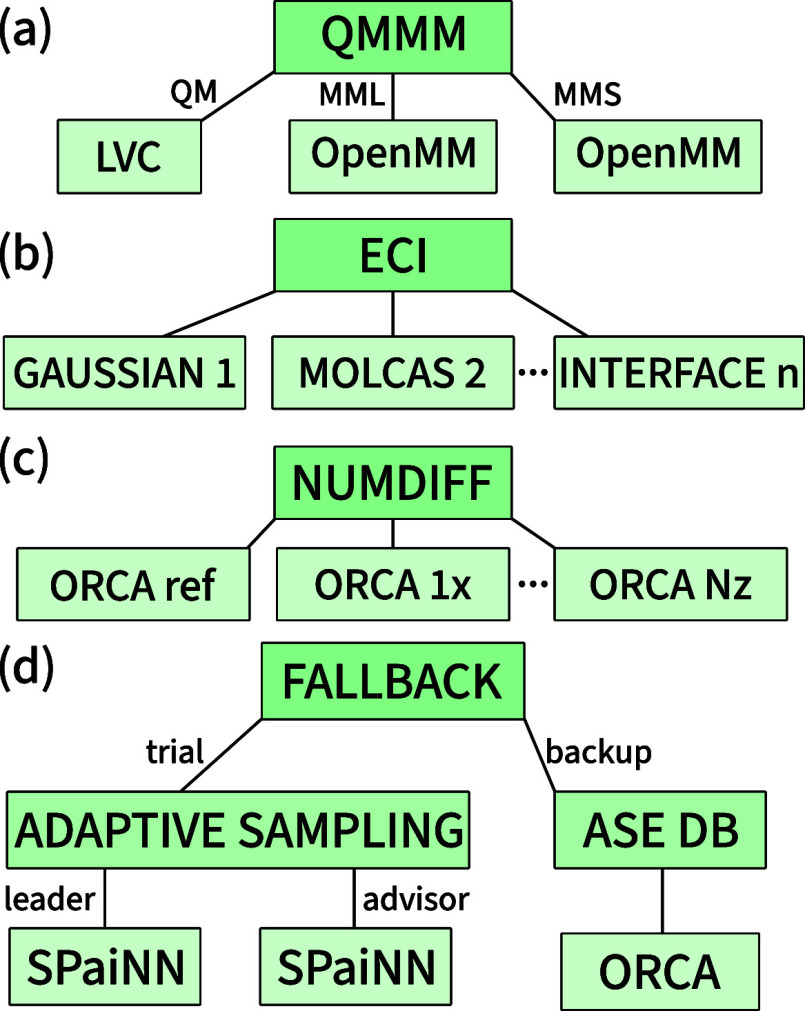
Examples of call trees for different hybrid interface schemes. (a) QM/MM using the hybrid interface labeled “QMMM” with LVC as the QM interface^55^ and OpenMM^64^ as the MM interface in a subtractive scheme (MML = MM of the entire “large” system; MMS = MM of the “small” QM region). (b) Excitonic configuration interaction^38^ using the ECI interface. Each fragment is handled by its own child interface. (c) Numerical differentiation using the NUMDIFF interface. It has a reference child interface (ORCA ref) and 3*N*_atom_ copies of that child interface for every displacement. (d) An example call tree for an adaptive sampling simulation using two SPaiNN^44^ machine learning models. If the two models produce disagreeing results on some time step, the ORCA interface is called to add data to an ASE^20^ database for further training.

#### 2.5.1 QM/MM with Electrostatic Embedding

The availability of hybrid interfaces has made it possible to completely redesign the implementation of QM/MM simulations with electrostatic embedding in a more convenient and flexible manner, see ref 55. In previous versions of SHARC, selected existing ab initio interfaces needed to be individually adapted to perform the MM and QM–MM interaction steps, in addition to the QM calculations. In contrast, the use of hybrid interfaces enables a dedicated, general QM/MM interface that is modular and largely agnostic to the programs used for the QM and MM regions. To run QM/MM simulations with electrostatic embedding, the QM/MM interface requires only two components: an MM interface capable of providing energies, gradients, and point charges for all atoms; and a QM interface that can accept these point charges, account for their influence on energies and wave functions, and return gradients (and possibly nonadiabatic couplings) with respect to the point charge positions. Any MM and QM interfaces meeting these requirements can be paired by means of the QM/MM interface. An example of a QM/MM call tree, using the linear vibronic coupling interface^55^ as QM interface, and OpenMM^64^ as the MM interface is sketched in Figure 2a.

Several other generic QM/MM implementations for nonadiabatic dynamics simulations were published in recent years, making a comparison with our framework expedient. Examples of such QM/MM implementations include a Gaussian/Tinker-based polarizable scheme in Newton-X,^47^ an Amber interface to NEXMD,^49^ the modularly designed, I/O-free INAQS scheme,^48^ a modular, file-based implementation in JADE,^65^ a modular high-performance implementation in ChemShell,^50^ another modular implementation in Cuby,^54^ and a flexible, file-based implementation in COBRAMM.^66^ Most of these QM/MM schemes were implemented in a file-I/O-based manner,^47,49,65,66^ due to a focus on ab initio electronic structure packages for the QM region. Other schemes have limited nonadiabatic capabilities^49,50^ and focus more on adiabatic dynamics. In terms of modularity and communication philosophy, the INAQS scheme^48^ and Cuby^54^ are closest to our implementation. However, our presented interface framework is the only scheme that combines nonadiabatic dynamics, I/O-free communication, multiple choices for the QM and MM interfaces (including fast analytical, vibronic coupling, and machine learning models models), integrated support for simulation setup, and the possibility to integrate QM/MM computations with other functionality like machine learning data acquisition or excitonic models.

#### 2.5.2 Excitonic Configuration Interaction (ECI)

ECI^38^ is a fragment-based electronic structure method designed to calculate energies of multichromophoric systems. It constructs an effective Hamiltonian from separate calculations for each monomer. This approach, and many related excitonic models,^67–70^ enables a description of excited states of extended systems with linear to quadratic scaling, which is much more efficient than any electronic structure calculation for the entire system. The ECI method was previously used to compute a Mg^2+^ coordinating guanine quadruplex,^38^ a chain of up to 32 boron-dipyrromethene (BODIPY) molecules, and a two-dimensional layer of up to 100 nitrogen-doped peri-xanthenoxanthene molecules with a total of 43,600 basis functions.^71^

The ECI method is agnostic to the specific electronic structure method used for each of the monomers, requiring only their energies and density matrices in terms of an atomic orbital basis set, which makes it highly flexible. Due to the modularity and flexibility of the ECI method, it is ideally suited to be implemented in the framework of an hybrid interface, allowing for seamless integration of different electronic structure calculations coming from different methods and softwares. An example of a interface call tree for ECI is shown in Figure 2b, where monomer 1 is computed using TD-DFT in GAUSSIAN,^72^ monomer 2 by CASSCF using OpenMOLCAS,^73^ and additional monomers through any other chosen interfaces. The only requirement of the child interfaces is to be capable of returning energies and density matrices.

#### 2.5.3 Numerical Differentiation

Using an hybrid interface it is possible to perform numerical differentiation in a flexible manner. Figure 2c shows a call tree for a newly developed numerical differentiation interface. Using Cartesian displacements of all atoms, this interface can compute gradients, nonadiabatic coupling vectors,^58^ and derivatives of spin–orbit coupling and dipole moment matrices. The interface can compute these derivatives in two different ways. In the simpler approach, *adiabatic* quantities are directly differentiated. Alternatively, the interface can use wave function overlaps to define *diabatic* states (that coincide with the adiabatic states at the reference geometry), which are then differentiated.

The Hamiltonian matrix at a displaced geometry is defined as **H**(*R⃗* ± δ*R⃗*_*ad*_) = **H** ^±*ad*^, where *R⃗* is the current geometry and *R⃗_ad_* is a unit displacement vector for atom *a* and direction *d*. This matrix is diagonal and contains the adiabatic energies. Likewise, **S**(*R⃗*, *R⃗* ± δ*R⃗_ad_*) = **S** ^±*ad*^ contains the wave function overlaps *S*_*ij*_^±*ad*^ = ⟨Ψ_*i*_(*R⃗*)|Ψ_*j*_(*R⃗* ± δ*R⃗_ad_*)⟩ between the adiabatic states at the reference and displaced geometries. Then, the *adiabatic* differentiation for gradients is simply done as

1 $$\frac{\partial E_{i}}{\partial R_{\mathrm{ad}}}=\frac{H_{\mathrm{ii}}^{+\mathrm{ad}}-H_{\mathrm{ii}}^{-\mathrm{ad}}}{2\delta }$$

and for nonadiabatic coupling vectors as^58^

2 $$\left\langle \Psi_{i}|\frac{\partial \Psi_{j}}{\partial R_{ad}}\right\rangle =\frac{S_{\mathrm{ij}}^{+ad}-S_{\mathrm{ij}}^{-ad}}{2\delta }$$

Alternatively, the *diabatic* numerical differentiation for gradients follows

3 $$\frac{\partial E_{i}}{\partial R_{\mathrm{ad}}}=\frac{1}{2\delta }{\left(S^{+\mathrm{ad}†}H^{+\mathrm{ad}}S^{+\mathrm{ad}}-S^{-\mathrm{ad}†}H^{-\mathrm{ad}}S^{-\mathrm{ad}}\right)}_{\mathrm{ii}}$$

while nonadiabatic couplings are obtained from

4 $$\left\langle \Psi_{i}|\frac{\partial \Psi_{j}}{\partial R_{ad}}\right\rangle =\frac{1}{2\delta \Delta E_{\mathrm{ij}}}{\left(S^{+ad†}H^{+ad}S^{+ad}-S^{-ad†}H^{-ad}S^{-ad}\right)}_{\mathrm{ij}}$$

where Δ*E*_*ij*_ is the adiabtic energy gap between states *i* and *j* evaluated at the reference geometry *R⃗*. The diabatic differentiation scheme is more elaborate, but it produces more robust derivatives in situations where significant state mixing occurs between the reference geometry and the displaced ones.

#### 2.5.4 Active Learning

Generating a training data set that adequately samples a large region of the conformational space of a molecule is a resource-intensive task, related to the computational cost of quantum chemical reference calculations. Active learning^74^ is able to reduce the number of data points required to train machine learning models by selectively sampling regions of high uncertainty. Starting from an initial training set, two or more preliminary models are trained and used for nonadiabatic dynamics—here, surface hopping trajectories. If these models’ predictions differ significantly at any time step, the geometry is added to the training set.^32^ Within the new interface framework, an active learning workflow can be set up using a three-level interface call tree like the one shown in Figure 2d. This tree contains different components: a fallback interface, an adaptive sampling interface, an interface for ASE^20^ databases, interface instances for the SPaiNN^44^ machine learning model, and an interface to provide electronic structure results for training, here the quantum chemistry software package ORCA.^75^

The fallback interface is a hybrid interface that uses two children, called the “trial” and “backup” interfaces. In each time step, first the trial interface is called and if it completes successfully, its results are simply passed on to the caller of the fallback interface. If the trial child returns an error (by raising an exception), the fallback interface would use the backup interface to obtain all required results for the current time step. The fallback interface can be set up so that a certain number of consecutive failures will terminate the trajectory.

In the illustrated example, the trial interface is the “adaptive sampling” interface. In turn, this interface is also hybrid with one “leader” child and at least one “advisor” child; here, both leader and advisor are instances of the SPaiNN interface, which have loaded different models for the same molecule. At each time step, the results of the advisor interfaces are compared to those of the leader. If they are consistent, the leader’s results are passed on, ensuring consistency in the PESs. However, if the results from the advisor deviates from those of the leader by more than a predefined threshold, an exception is raised. Here, the adaptive sampling interface allows flexibly customizing how to quantify the deviation of leader and advisors. For each attribute of the QMout object (see above), a separate threshold can be defined. Various error functions (e.g., mean absolute deviation, root-mean-square deviation) and the possibility to load custom error function code are also provided.

The exception raised in the trial child would trigger the call of the backup child of the fallback interface – here, the ASE database interface. The ASE database hybrid interface simply passes all requests onto its only child, which performs the actual computations and returns the results. The ASE database interface writes all results received from its child, plus the current geometry, to a database (using the ASE package^20^) before they are passed on to the caller of the ASE database interface.

Having the database functionality as a separate hybrid interface enables users to easily choose which data to store. For example, in a QM/MM scheme one could choose to store the full-system results of the entire QM/MM calculation (by setting the database interface as parent of the QM/MM interface) or only the results of the QM subsystem (by setting the database interface as the QM child of the QM/MM interface). Likewise, in an ECI calculation, one could store the results of each fragment in a separate database, rather than storing the full-system results. In the active learning scheme in Figure 2d, only the results of the ORCA calculations are stored, but not the results of the machine learning interfaces, because the database interface only stores the results coming from its own child. We also note that the dynamics driver itself will only ever see the results coming from the parent interface, so that only the results for the entire system are stored by default (by the driver). The child interfaces of hybrid interfaces do not automatically store their results for all time steps, but only files needed for restart/wave function overlaps with the previous time step. Hence, hybrid interfaces would in principle produce the same amount of dynamics simulation data as using a regular interface.

We anticipate that simple single-child hybrid interfaces—like the database interface—can be used in the future to selectively and flexibly manipulate the electronic structure data according to the user’s demands, without changing the dynamics driver code. For example, a single-child hybrid interface could be used to add a bias potential to all electronic energies and gradients, facilitating accelerated dynamics (e.g., umbrella sampling).^76^ One could also multiply the spin–orbit couplings by a factor in order to investigate the dependence of intersystem crossing time scale and yield on the magnitude of these couplings. Likewise, one could set the gradients on certain atoms to zero in order to anchor them in space, e.g., as done in some QM/MM schemes.^66^

## 3 Computational Details

The capabilities of the new interface framework have been already exploited in recent LVC/MM dynamics simulations^55,77^ and ECI energy calculations.^38,71^ Here, we further demonstrate its versatility through two additional examples. One is a minimum-energy conical intersection optimization with TDDFT using nonadiabatic coupling vectors from the numerical differentiation interface. In the second example, we create an active learning workflow using hybrid interfaces to train a machine learning model, subsequently used in TSH simulations. For both examples, all relevant input files are provided in a Supporting file archive, whose content is briefly explained in the Supporting Information in Section S1.

As the test system for both examples, we use 4-(Dimethylamino)­benzonitrile (DMABN, see Figure 3), which has been proposed as the “molecular Tully model II”.^78,79^ It features two energetically close electronic states and upon photoexcitation, the conical intersection between them is traversed repeatedly. This molecule is an attractive model for our study due to its well-documented excited-state dynamics, its moderate size, relatively low computational cost when treated with TD-DFT, and the absence of ground-state relaxation, which further supports TD-DFT as a suitable method for dynamics.

**Figure 3:**
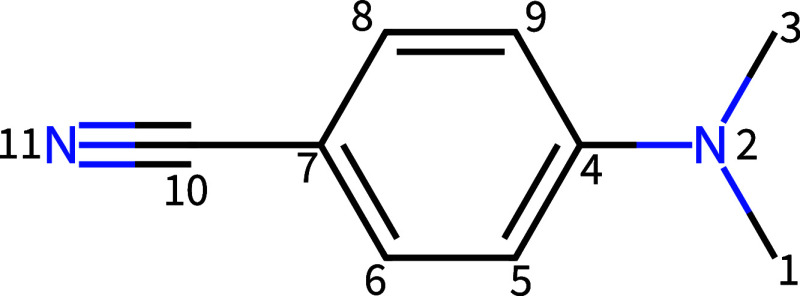
Structure and atom numbering of 4-(Dimethylamino)­benzonitril (DMABN).

All electronic structure calculations were performed using (TD-)­DFT with ORCA 6.0.1,^75^ with the ωB97X-D3 functional^80^ and the def2-SV­(P)^81^ basis set, along with RIJCOSX,^82^ and the Tamm–Dancoff approximation.^83^ The calculations are done in gas phase and included three singlet states. For the conical intersection optimization, the “verytightscf” settings and the “defgrid3” integration grid were used. For the trajectories and trainings data points in the active learning workflow, default convergence thresholds and integration grids were employed.

### 3.1 Minimum-Energy Conical Intersection Optimization

The minimum-energy conical intersection optimization was started from a random snapshot from one of the ML-based trajectories described below (at that snapshot, the S_2_–S_1_ energy gap was 0.25 eV). The S_2_/S_1_ minimum-energy conical intersection was first approximately optimized using the penalty function approach of Levine et al.,^84^ using the parameters σ = 3.5 and α = 0.02, as described in that publication, and three states. Note that with this approach, the minimum-energy conical intersection will not be reached exactly, but a small energy gap typically remains, depending on the conical intersection topology and the chosen σ and α. From the final geometry, we continued to optimize toward the minimum energy conical intersection using the algorithm of Bearpark et al.,^85^ employing numerical gradients and nonadiabatic coupling vectors as computed with the numerical differentiation interface with diabatic differentiation (eqs 3 and 4).

### 3.2 Active Learning

For the active learning workflow, the ground state equilibrium geometry of DMABN was first optimized with DFT as described above. From the obtained frequencies and normal modes, a harmonic Wigner distribution was defined and 1000 geometries were sampled. At each of these geometries, a single-point calculation with the ASE database and ORCA interfaces was performed. The results—1001 geometries (equilibrium plus 1000 Wigner samples) with energies and gradients for three singlet states—were stored in an ASE database. Using this initial data set, two SPaiNN models were trained with different random data splits, maintaining a train/validation/test ratio of 8:1:1.

In the first round of adaptive training, based on the first 500 Wigner samples, a total of 1000 trajectories were launched, each sample separately starting in S_1_ and S_2_ to ensure that both states are sampled adequately. The trajectories were propagated with a 0.5 fs time step for up to 1000 fs. Curvature-driven couplings^57^ were used to propagate the electronic wave function, because the possible alternatives available within SHARC are not compatible between ORCA and SPaiNN: TD-DFT in ORCA cannot provide nonadiabatic couplings, whereas SPaiNN cannot provide wave function overlaps^58^ that could be used with a local diabatization propagator.^86^ The trajectories were run using the interface tree displayed in Figure 2d, with a mean absolute error threshold of 0.8 m*E*_*h*_ (22 meV) between leader and advisor for the energies of all three states in the adaptive sampling interface. A trajectory was aborted if exceptions are raised by the adaptive sampling interface in two consecutive time steps. In this way, the 1000 trajectories of the first round produced 3217 new data points.

For the second round of training, two new SPaiNN models were trained with all 4218 data points (equilibrium plus Wigner samples plus points from the first round). The second round was carried out with the same settings as the first one, producing additional 3050 data points.

In a third and final round, with the entire data set of 7268 points, a final SPaiNN model was trained, using the same parameters. Starting in S_2_, 500 trajectories were launched using this final SPaiNN model. As we did not intend to collect any additional training data points at this stage, these final trajectories were propagated using only the SPaiNN interface rather than the entire interface tree in Figure 2d, which is computationally more efficient. As a reference, we also propagated 50 trajectories using TD-DFT from ORCA during 300 fs, with otherwise identical settings as in the machine learning trajectories.

## 4 Results and Discussion

### 4.1 Minimum-Energy Conical Intersection Optimization

Minimum-energy conical intersections are important for understand photoinduced relaxation mechanisms. They can be optimized in different ways: One is the penalty function algorithm by Levine et al.,^84^ which only uses gradients but no nonadiabatic couplings, making it amenable to many TD-DFT implementations. This algorithm, however, usually does not find the lowest energy point in the degenerated seam. A second, somewhat more rigorous optimization approach is the projected-gradient technique of Bearpark et al.,^85^ which can locate the lowest energy point but requires nonadiabatic coupling vectors. Here, we optimize the S_2_/S_1_ conical intersection of DMABN with both approaches to show that the new numerical differentiation interface delivers accurate nonadiabatic coupling vectors from TD-DFT.

The results of the two optimizations of the S_2_/S_1_ minimum-energy conical intersection are summarized in Table 1. Both optimized geometries are compared in Figure 4; their RMSD is 0.012 Å. As could be expected, the conical intersection optimization with the penalty function method was considerably cheaper than the method using numerical nonadiabatic couplings, with very similar results. There might be other systems and other minimum-energy conical intersections where the two methods will differ significantly more, e.g., in strongly sloped conical intersections. However, in this example the focus is not to promote the numerical approach for production calculations, but to demonstrate that the numerical differentiation scheme produces correct nonadiabatic coupling vectors. Given that the numerical optimization produced a smaller energy gap, we deem this demonstration successful. Our results also confirm that the employed nonadiabatic coupling vectors—derived simply from differentiation of overlaps computed from TD-DFT amplitudes—are equivalent to nonadiabatic coupling vectors properly derived from quadratic response theory.^87^

**Figure 4:**
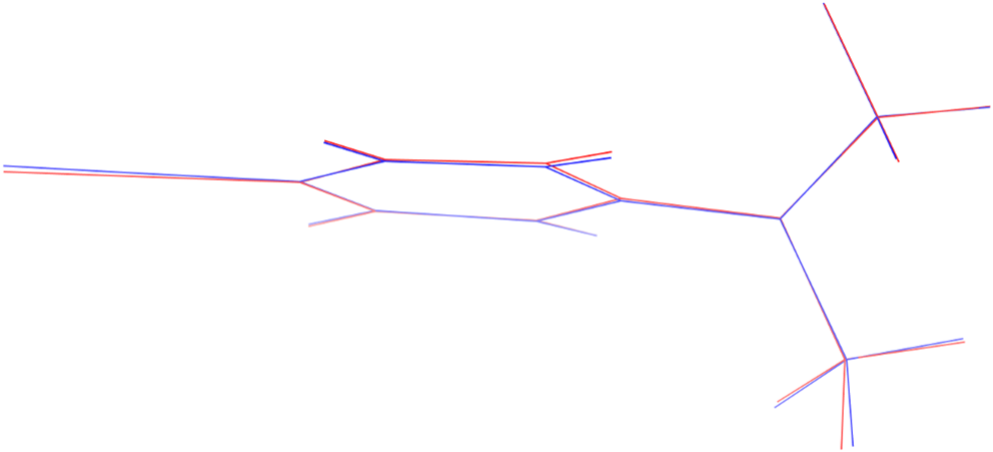
Overlay of the two optimized structures of the S_2_/S_1_ minimum-energy conical intersection of DMABN, obtained using (i) the penalty function method with analytical gradients in blue and (ii) the projected-gradient method with numerical gradients and nonadiabatic coupling vectors in red.

**Table 1:** Results of the Optimization of the S_2_/S_1_ Minimum-Energy Conical Intersection of DMABN, using (i) the Penalty Function Method with Analytical Gradients and (ii) the Projected-gradient Method with Numerical Gradients and Nonadiabatic Coupling Vectors

	penalty function method	numerical method
*E* ^total^(*S*_2_)/*E*_*h*_	–457.794 075	–457.794 173
*E* ^rel^(*S*_2_)/*E*_*h*_	0.0	0.000 098
*E* ^rel^(*S*_2_)/meV	0.0	2.7
Δ*E*(*S*_2_–S_1_)/*E*_*h*_	0.000 387	0.000 048
Δ*E*(*S*_2_–S_1_)/meV	10.5	1.3
Iteration count	38	65
Wallclock time^a^	1 h on 4 CPU cores	19 h on 21 CPU cores

a on an AMD EPYC 7502 processor with 3.3 GHz.

To showcase that similar optimizations of minimum-energy conical intersections could also be carried out with the new interface framework with many other electronic structure methods and packages, we present numerical computations of nonadiabatiac coupling vectors in Figure S2. We computed the S_1_/S_2_ nonadiabatic coupling vector at the optimized geometry shown in Figure 4. We employed TDA-ωB97X^88^/def2-SV­(P) in ORCA^75^ and Gaussian,^72^ TDA-ωB97X^88^/DZP in AMS ADF,^89^ TDA-PBE^90^/def2-SVP in NWChem,^91^ ADC(2)/def2-SV­(P) in Turbomole,^92^ and compared to analytical coupling vectors^47^ with XMS-CASPT2/cc-pVDZ in OpenMolcas.^73^ The results in Figure S2 show that all six calculations produce consistent nonadiabatic coupling vectors, even though vastly different methods were employed. Hence, the interface framework provides a large degree of interoperability that is useful for many users.

### 4.2 Surface Hopping with Active Learning

Time-resolved electronic populations of the S_2_, S_1_, and S_0_ states obtained from TSH simulations are presented in Figure 5 for reference TD-DFT trajectories (panel a) and for the three sets of machine-learning trajectories, after initial training (b) and after one (c) or two (d) rounds of active learning. The shown populations are classical populations, i.e., fractions of trajectories in each active state. The confidence intervals correspond to a 95% confidence level and were computed as $P\pm 1.96\sqrt{\frac{P\left(1-P\right)}{N_{\mathrm{traj}}}}$ , where *P* is the current population.

**Figure 5:**
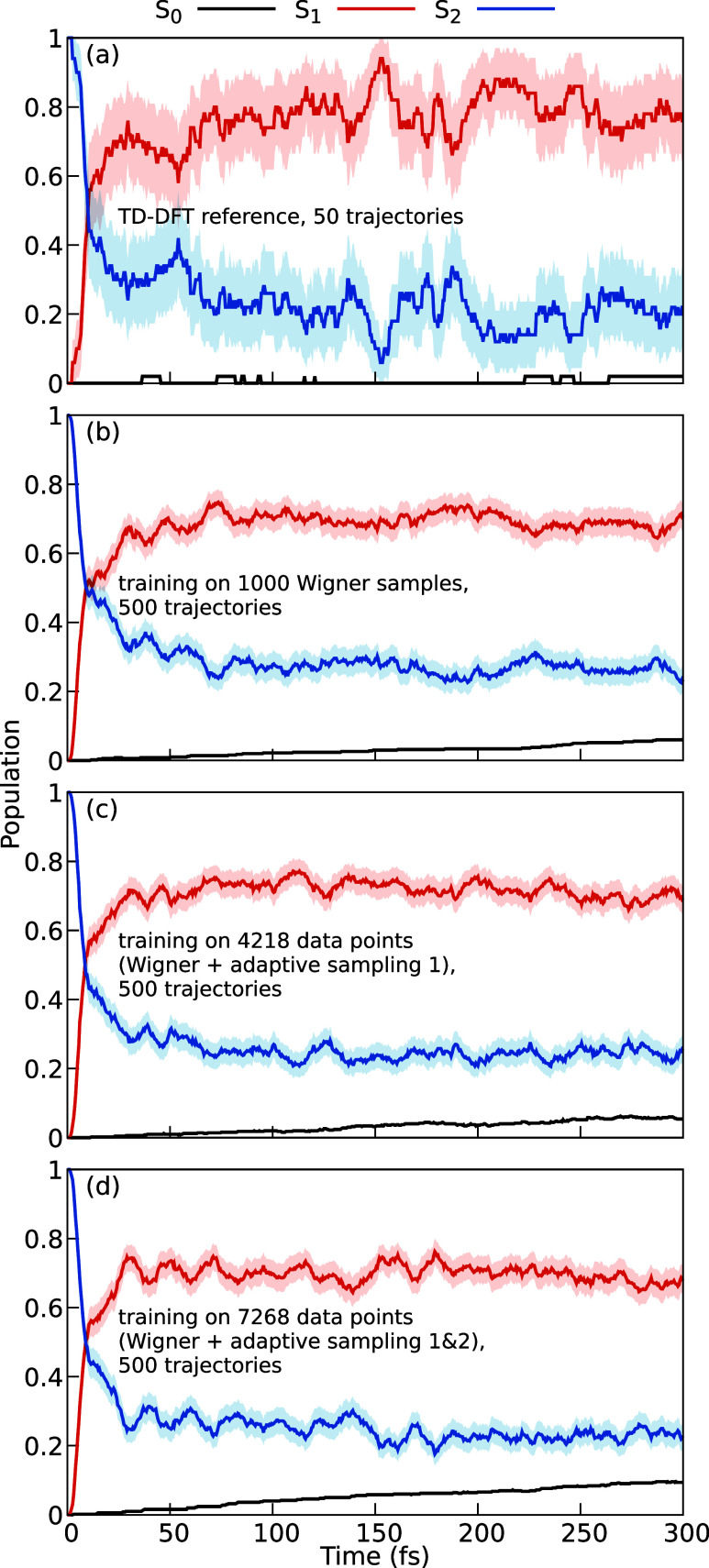
Populations of DMABN trajectories started from S_2_ with TD-DFT and different SPaiNN models including 95% confidence intervals (shaded areas). (a) TD-DFT reference, 50 trajectories. (b) Model trained on initial conditions, 500 trajectories. (c) Model trained with additional data from the first adaptive sampling run, 500 trajectories. (d) Model trained with additional data from the first and second adaptive sampling run, 500 trajectories.

As shown in Figure 5a, a large amount of the population initially excited to the S_2_ state decays quickly to the S_1_ state. Already after 10 fs, the S_1_ and S_2_ populations are of equal magnitude. Afterward, the S_1_ population increases slower, evidencing superimposed irregular oscillations between the S_1_ and S_2_ states that indicate that the respective conical intersection is relatively accessible from the S_1_ surface. After 300 fs, the S_1_ population has reached a value of 76 ± 12%. Due to the small number of trajectories (50) the confidence interval is relatively large.

The results obtained with the three machine learning runs are shown in panels b–d. As we ran 500 trajectories in each round, the confidence intervals are significantly narrower than with TD-DFT (panel a). Overall, the agreement of the populations with the TD-DFT reference is qualitatively good. All three models show equal S_1_ and S_2_ populations at 9.5 fs and S_1_ populations at 300 fs of 71 ± 4, 69 ± 4, or 68 ± 4%, respectively. These results agree within the confidence intervals with the TD-DFT values, showing that the rapidly developed machine learning models can effectively reproduce the electronic dynamics of DMABN. We note that the S_2_ to S_1_ population transfer might be somewhat slower and decay to S_0_ somewhat faster for the machine learning models than for TD-DFT. This is because curvature-driven surface hopping is very sensitive to the local curvature of the PES, which is more challenging to learn than the overall large-scale features of the PESs.

We also investigated the agreement of the nuclear dynamics between TD-DFT and machine learning. In Figure 6, we plot the temporal evolution of the dihedral angle between the aromatic ring and the dimethylamino group. The reference TD-DFT trajectories (panel a) show that, initially, the dihedrals are distributed closely around zero, as provided by the ground state Wigner distribution. Over time, the dihedrals change notably, leading to a bimodal distribution centered around ± 23 degrees with no trajectories remaining near zero. The machine learning trajectory swarms (panels b–d) start from the same Wigner distribution, but clear differences can be observed for the three rounds of active learning. The model trained only on the 1000 Wigner distribution samples exhibit essentially no torsion, so that the dihedrals are still distributed around zero degrees at 300 fs. The two models arising from active learning are much better. The final model (panel d) shows a clear bimodal distribution centered around ± 22 degrees. The satisfactory agreement to the TD-DFT reference evidence that active learning can effectively improve the fidelity of machine learning-predicted PESs.

**Figure 6:**
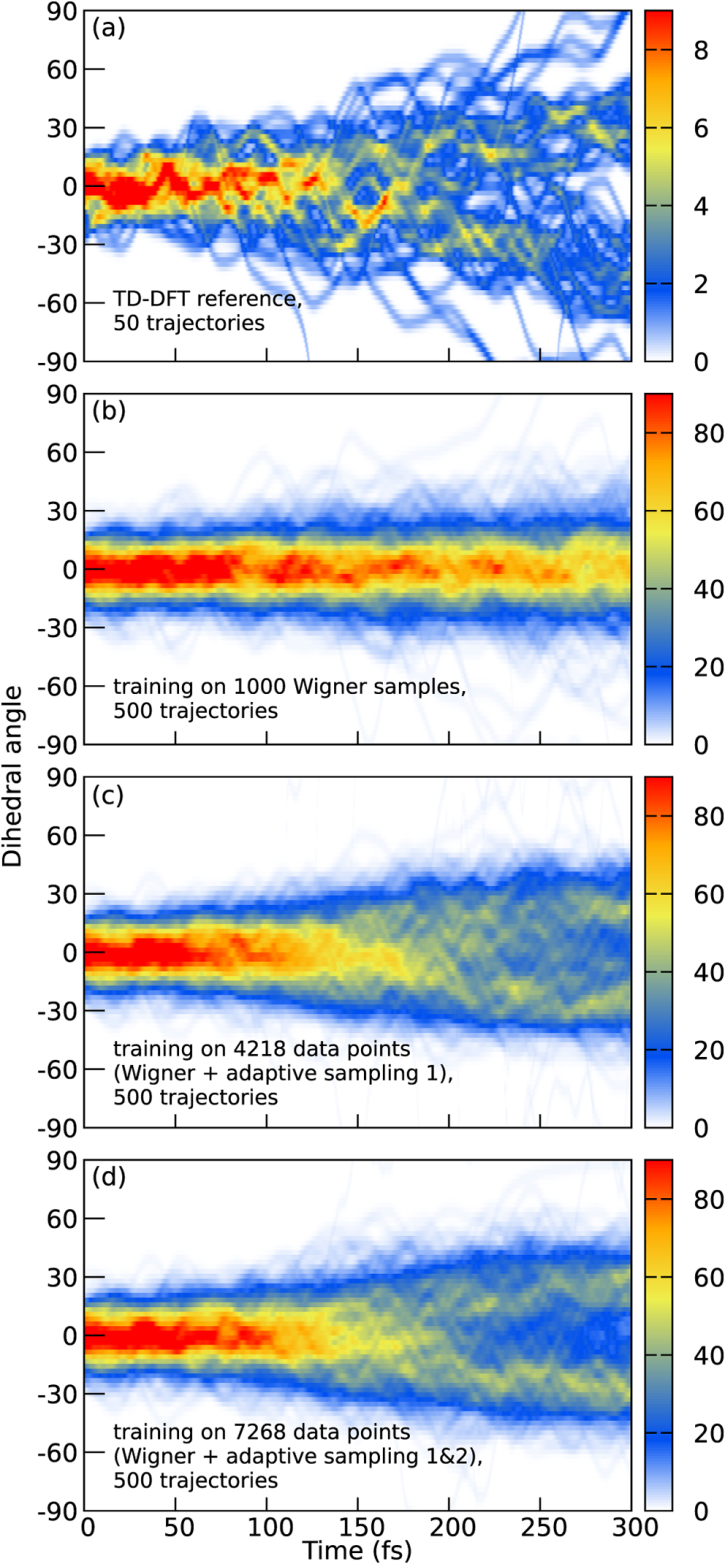
Dihedral angles of the ring-dimethylamino torsion using atoms 9–4–2–3 (see Figure 3). (a) TD-DFT reference from 50 trajectories, (b) SPaiNN model trained on initial conditions from 500 trajectories, (c) SPaiNN model trained on initial conditions plus first adaptive sampling run from 500 trajectories, (d) SPaiNN model trained on initial conditions plus first and second adaptive sampling run from 500 trajectories.

We emphasize that the presented example application serves primarily as a simple demonstration of how workflows can be efficiently encoded using the new hybrid interfaces, rather than a production machine learning surface hopping project. For a full-scale machine learning project, more attention should be paid to the initial sampling, the amount of new data points added per active learning round, and the filtering of data points from already-sampled regions. Nonetheless, the example clearly illustrates that the entire training data acquisition process can be achieved by simply running trajectories with the appropriate interface call tree.

Finally, we want to comment on the computational efficiency of SHARC 4 in conjunction with the class of fast (I/O-free) interfaces. The 500 final trajectories using a single SPaiNN model (Figures 5d and 6d) were run on an AMD EPYC 7502 processor (clock speed of 3.3 GHz). We obtained a total wall clock time per trajectory of about 170 s, which amounts to 83 ms per time step or 0.05 CPUh/ps with the used 0.5 fs time steps. For comparison, a trajectory running with the entire interface tree in Figure 2d (i.e., with active training) cost 171 ms per time step (neglecting any time steps where ORCA was called), due to the use of two SPaiNN models and the overhead from the fallback and adaptive sampling interfaces. One ORCA single point calculation (with three states and three gradients) took about 390 s. Hence, the ORCA computations to produce training data took less than 800 CPUh. The training of both SPaiNN models with the initial data set took about 10 GPUh, the training of both SPaiNN models after the first adaptive sampling run took about 42 GPUh, and the training of the last model after the second adaptive sampling run took about 27 GPUh on a Nvidia Tesla P100 GPU.

## 5 Conclusions

We have presented a novel software framework for electronic structure interfaces for nonadiabatic dynamics simulations using concepts of object-oriented programming. Within this framework, three base classes—for fast, ab initio, and hybrid interfaces—were introduced, providing general code for interfaces that communicate with different kinds of electronic structure models. The object-oriented design ensures that the code is maintainable, extendable, and reusable. The design and strict interface guidelines make it possible to develop new interfaces quickly without needing to fully understand all internal details. Efficient communication between the driver and interface ensures that fast electronic structure methods can be executed within memory, avoiding overhead caused by file I/O and reinitialization. Furthermore, the use of nested hybrid interfaces allows users to construct custom workflows, without the need to write any code. Although this framework was developed within the context of the SHARC surface hopping package^16^ (and is available under the GNU General Public License via GitHub^16^), it is principally standalone code that could easily be used by any nonadiabatic dynamics package. In the future, the interface framework can be used to enable efficient communication with high-performance quantum chemistry software (e.g., Fermions++^94^) or with novel approaches rooted in quantum computing,^95^ to incorporate umbrella sampling in excited states, or to develop different multiscale methods. We hope the design of the presented interfaces provides a useful reference for future developments in software targeting excited-state dynamics.

## Supplementary Material





## Notes

Explanations for input files for the adaptive sampling and conical intersection optimization examples, numerical differentiation computation of nonadiabatic coupling vectors with different methods/packages (PDF)
Input files for the adaptive sampling and conical intersection optimization examples (ZIP)
