# Supplementary material for: Efficient, Hierarchical, and Object-Oriented Electronic Structure Interfaces for Direct Nonadiabatic Dynamics Simulations
Source: J Chem Theory Comput. 2025 Sep 10;21(18):8994–9008. doi: 10.1021/acs.jctc.5c00878 (PMC12461921; doi:10.1021/acs.jctc.5c00878)
Supplement: Supplementary file 1 [file ct5c00878_si_001.pdf]

Supporting Information: Efficient, Hierarchical, and Object-Oriented Electronic Structure Interfaces for Direct Nonadiabatic Dynamics Simulations

Sascha Mausenberger,[†,‡] Severin Polonius,[†,‡] Sebastian Mai,[*,†] and Leticia González[*,†,¶]

†*Institute of Theoretical Chemistry, Faculty of Chemistry, University of Vienna, Währinger Straße 17, 1090 Vienna, Austria*

‡*Vienna Doctoral School in Chemistry (DoSChem), University of Vienna, Währinger Straße 42, 1090 Vienna, Austria*

¶*Research Platform on Accelerating Photoreaction Discovery (ViRAPID), University of Vienna, Währinger Straße 17, 1090 Vienna, Austria*

E-mail: sebastian.mai@univie.ac.at; leticia.gonzalez@univie.ac.at

Contents

S1 Adaptive Sampling example **S-4**

S1.1 Preparation of initconds S-5

S1.2 Setup trajectories S-5

S1.3 Adaptive sampling S-5

S1.4 Conical intersection optimization S-5

S2 Numerical computation of nonadiabatic coupling vectors **S-6**

S1 Adaptive Sampling example

```
SI/
└── adaptive_sampling
    ├── KEYSTROKES.setup_init
    ├── KEYSTROKES.setup_traj
    ├── KEYSTROKES.excite
    ├── initcond/
    │   ├── ASE_DB.template
    │   ├── ASE_DB.resources
    │   ├── run.sh
    │   ├── QM.in
    │   └── QM/
    │       ├── ORCA.resources
    │       └── ORCA.template
    └── trajectory/
        ├── input
        ├── veloc
        ├── run.sh
        ├── geom
        └── QM/
            ├── FALLBACK.template
            ├── FALLBACK.resources
            ├── trial_interface/
            │   ├── ADAPTIVE.resources
            │   ├── ADAPTIVE.template
            │   ├── model1/
            │   │   ├── SPAINN.template
            │   │   └── SPAINN.resources
            │   └── model2/
            │       ├── SPAINN.template
            │       └── SPAINN.resources
            └── fallback_interface/
                ├── ASE_DB.template
                ├── ASE_DB.resources
                └── QM/
                    ├── ORCA.template
                    └── ORCA.resources
└── numdiff
    ├── NUMDIFF.resources
    ├── NUMDIFF.template
    ├── run_EXTORCA.sh
    ├── geom.xyz
    ├── orca.inp
    ├── otool_external.inp
    └── QM
        ├── ORCA.template
        └── ORCA.resources
```

Figure S1: Contents and file structure of the `si.tar.gz` file that accompanies this SI. The folder `adaptive_sampling` contains files related to the adaptive sampling example. `adaptive_sampling/initcond` contains resource and template files used to calculate data for the initial database with an example `QM.in` file and an example run script `run.sh`. The folder `adaptive_sampling/trajectory` contains input files that were used to run trajectories for active learning. The folder `numdiff` contains resource, template, and ORCA input files used to run a MECI optimization.

S1.1 Preparation of initconds

First, a frequency calculation was performed in ORCA for the optimized ground-state structure of DMABN using the wB97X-D3/def2-SV(P) level of theory. Then, initial coordinates were generated from 1000 Wigner samples. The results from these Wigner samples were then used to create a database for training purposes. The `KEYSTROKES.setup_init` file used to setup the initial conditions is attached in `si.tar.gz` in the `adaptive_sampling` directory.

The `ASE_DB` hybrid interface was used in combination with the `ORCA` interface to collect the data for the latter training. All input files are collected in the `si.tar.gz` archive in the directory `adaptive_sampling/initcond/`.

S1.2 Setup trajectories

Using the `initconds` file from the previous section, excited-state initial conditions were generated with the `excite.py` script from SHARC. Next, trajectories were prepared using the `setup_traj.py` script, also from SHARC. The file `KEYSTROKES.setup_traj`, which was used to configure the excited-state initial conditions, is included in the `si.tar.gz` archive located in the `adaptive_sampling` directory.

S1.3 Adaptive sampling

The trajectories generated in the previous section were used for the adaptive sampling runs. All resource and template files needed are included in `si.tar.gz` in the directory `adaptive_sampling/trajectory/QM/`.

S1.4 Conical intersection optimization

The folder `numdiff` in `si.tar.gz` contains input files to optimize the S_1/S_2 conical intersection using the ORCA external optimizer, SHARC, and another instance of ORCA that performs the TDDFT calculations.

S2 Numerical computation of nonadiabatic coupling vectors

Figure S2 compared the nonadiabatic coupling vectors computed for different methods through the numerical differentiation interface (with diabatic differentiation) with different quantum chemistry backends. These vectors were computed via single-point calculations at the optimized S_1/S_2 minimum-energy crossing point.

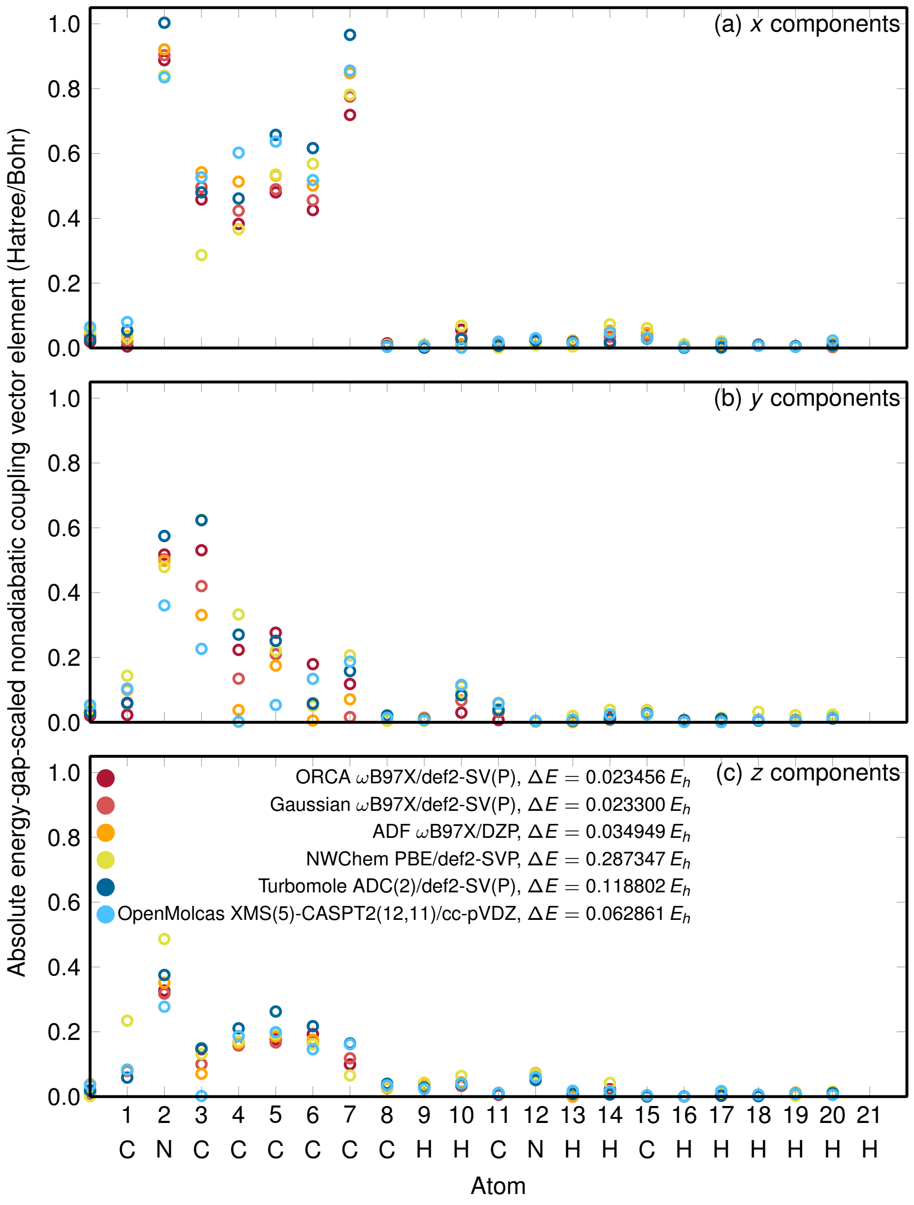

Figure S2: Plot of the absolute magnitudes of the energy-gap-scaled nonadiabatic coupling vectors $\Delta E_{S_1 S_2} \left\langle \Psi_{S_1} \middle| \frac{\partial \Psi_{S_2}}{\partial R_{Ai}} \right\rangle$, with atom number A (bottom axis) and Cartesian component i (panels a–c).

Note that the different quantum chemistry programs employed—ORCA,[75] Gaussian,[72] AMS ADF,[90] NWChem,[92] Turbomole,[93] and OpenMolcas[94]—do not all have the method employed in the main manuscript (ωB97X-D3[80]/def2-SV(P)[81]) available. Hence, for Figure S2, we have employed the ωB97X[89] functional instead, which is available in ORCA, Gaussian, and AMS ADF. As AMS ADF does not use GTO-style basis functions, we picked the DZP basis set instead. For NWChem, using the range-separated ωB97X was not possible with our installation, so we decided to switch to a completely different functional (PBE[91]) to check how robust the computed coupling vectors are. Furthermore, in NWChem the def2-SV(P) basis set is located in the same file as def2-SVP, which lead to problems with the wave function overlap computations; hence, we used def2-SVP instead. Turbomole was used to the numerical nonadiabatic coupling vector at the ADC(2)/def2-SV(P) level of theory. OpenMolcas was used to compute the analytical[47] nonadiabatic coupling vector at the XMS-CASPT2/cc-pVDZ level of theory, using 5 states for state averaging and the XMS procedure.

The results in Figure S2 show that all six employed methods produce qualitatively consistent nonadiabatic coupling vectors between the S_1 and S_2 states. Note that, due to the use of different levels of theory than in the main manuscript, the geometry does not correspond to the conical intersection. Thus, it is more useful to compare the obtained nonadiabatic coupling vectors with each other. The results from ORCA and Gaussian agree the closest with each other, as they were computed at the same level of theory.

Nonetheless, the figure shows that with the numerical differentiation interface, consistent nonadiabatic coupling vectors are obtained for the same state pair for a large variety of different electronic structure programs and methods. The largest deviation between two nonadiabatic coupling vectors is found between the ORCA calculation and the OpenMolcas one, with an angle of about 25° between the vectors. The other pairwise angles between the obtained vectors are between 7 and 24°.
